# Streaming *Diversité*: Exploring representations within French-language scripted series on Canadian SVOD services

**DOI:** 10.1177/13548565241270691

**Published:** 2024-08-13

**Authors:** Stéfany Boisvert

**Affiliations:** School of Media, 14845 Université du Québec à Montréal, Canada

**Keywords:** Canada, SVOD services, diversity, streaming, Quebec, French, original content, scripted series

## Abstract

Canadian subscription-video-on-demand (SVOD) services have commissioned French-language ‘original’ content to attract local audiences. ICI TOU.TV, Club Illico and Crave have indeed commissioned more than a hundred French-language scripted series, mostly produced in the Quebec province. However, the current state of research only marginally documents these services. Even in Canada, most research focus on US-owned streaming giants such as Netflix and Amazon, thus providing little information on Canadian national SVOD services, and their affordances in terms of storytelling and representation. Current research also completely overlooks French-language original content. This paper therefore discusses the results of the very first research project to specifically focus on the production of original French-language series for Canadian streaming services. After reviewing all original (scripted and unscripted) French-language content available on Canadian-owned SVOD services, a textual analysis of more than 40 scripted series has been conducted, which led to intricate insights regarding prevailing narrative trends and characteristics of main and secondary characters. In so doing, the objective was also to determine the level of diversity included within this so-called original content. In a context characterized by an unprecedented proliferation of scripted series, it indeed becomes crucial to ascertain whether a greater quantity of productions necessarily leads to a greater diversity in representation, that is, the inclusion of a ‘multiplicity of forms’, and an equitable plurality of cultural expressions and identities. This research produced several findings that testify to a significant inclusion of sexual, gender, and racial diversity, as well as a noticeable trend towards intersectional representation. Yet, the analysis also led to identify persistent issues, such as the qualitative marginalization of non-normative characters (queer, BIPOC, with disability, etc.), as they mostly are relegated to supporting roles. These findings therefore call for a nuanced assessment of the ‘progress’ in representation on streaming services.

## Introduction

Since 2010, the Canadian audio-visual industry has adapted to new modes of distribution by creating national subscription-video-on-demand (SVOD) services. Yet this shift towards digital streaming is not just an adaptation to new modes of distribution; it is also a strategic response that reflects deep-seated concerns about the potential loss of domestic audiences to colossal transnational platforms. In Canada, including Quebec – where French predominates – almost half of the population (48%) subscribes to Netflix. Moreover, Amazon Prime Video and Disney+ occupy the market’s second and third ranks, signaling a diminishing share for local streaming services (Académie de la transformation numérique, 2023: 16).

In the face of this growing threat from foreign platforms, and motivated by a commitment to cultural sovereignty, one of the most important strategies has consisted of creating ‘original content’ for local SVODs. Canadian streaming services ICI TOU.TV, Club Illico and Crave have thus commissioned more than one hundred French-language scripted series, which have mainly been produced in the Quebec province.^1^

Producing original shows for streaming services not only changes the way audio-visual content is distributed: it also influences the nature of content itself. This is particularly salient in a milieu where local media industries navigate the erosion of protectionist policies, all while striving to optimize the discoverability of local content – its *visibility*, *findability*, and *accessibility* online McKelvey and Hunt, 2019; cf. also Canada Media Fund, 2016). Furthermore, the current state of the global audio-visual sector is characterized by an overabundance of content (Euvrard et al., 2018: 5). This profusion puts additional strain on national media industries as both production funding and audience attention are dispersed across an ever-growing number of productions (Euvrard et al., 2018). In this context, documenting and scrutinizing the nascent practices of original content creation within French-language Canadian OTT services becomes essential to better understand the contemporary dynamics of media production in the streaming era.

However, the existing body of research provides only a cursory examination of Canadian SVOD services. Even in Canada, research mostly focuses on the biggest streaming platforms of US origin like Netflix (Claus, 2017; Fragata and Gosselin, 2018; Schnitzer, 2019; Wagman, 2017; Zboralska and Davis, 2017), therefore leaving a significant knowledge gap about the creative practices related to Canadian SVOD services, in terms of storytelling and representation. Particularly neglected is the substantial body of French-language original content (Boisvert, 2019a; Boisvert et al., 2024).^2^

To address this scholarly gap, a research initiative – the very first of its kind – was launched in 2020, dedicated to cataloguing and analyzing original French-language series on Canadian streaming services. A pivotal goal of this project is to evaluate the diversity encapsulated within these so-called ‘original’ productions. In a context characterized by an unprecedented proliferation of scripted series, it becomes crucial to ascertain whether a greater quantity of productions necessarily leads to a greater diversity in representation. The article will provide an overview of this quantitative and qualitative analysis of French-language original scripted content, highlighting several trends and issues regarding the inclusion of diversity in Canadian online series. Namely, I argue that there is significant inclusion of sexual, gender, and racial diversity in scripted series, and a noticeable trend towards intersectional representation. However, the analysis also reveals persistent challenges, including the qualitative marginalization of non-normative characters and the near invisibility of certain social groups.

## Methodology

For my research project, a comprehensive review of all original (scripted and unscripted) French-language content available on Canadian-owned SVOD services (distributed from December 2010 to February 2024^3^) was first undertaken. Based on these findings, a database, updated quarterly, was created. In addition to tagging each content as ‘scripted’ or ‘unscripted’, the database provides specific details such as the synopsis, cast and crew, central themes, and significant dialogues. This rich repository facilitates extensive cross-referencing and analysis.

Of all the original content listed for this research, 102 productions fall into the broad category of ‘scripted content’. In order to focus more specifically, for the present article, on diversity in scripted series, a more detailed textual analysis was subsequently conducted on approximately 40 of these scripted series,^4^ which led to intricate insights regarding prevailing narrative trends, as well as a synthesis of the main characteristics of primary and secondary characters. This research therefore combines a macro and micro approach, as well as a quantitative and qualitative analysis, to better understand the kinds of content French-language Canadian streaming services commission amidst the competition posed by international platforms.

This article focuses on the three most popular SVOD services known for their original French-language scripted series: ICI TOU.TV, Club Illico, and Crave. Established by the French-language public broadcaster (Société Radio-Canada), ICI TOU.TV was inaugurated in 2010, predating Netflix Canada by a few months. Originally operating as a free ad-supported Web site, ICI TOU.TV evolved into a *hybrid portal* (Lobato, 2019; Lotz, 2017, 2018) in 2014, offering a basic catalog free of charge as well as a premium subscription-based section known as ‘the Extra’. Club Illico was then launched by cable operator Videotron in 2013, signaling its intention to dominate the local streaming market by becoming the ‘Québécois Netflix’. A year later, Bell Media introduced Crave, a bilingual SVOD service catering to Canada’s dual official languages. Although Crave primarily featured English-language series and films, it expanded in 2020 to include French-language originals. As of 2024, these services collectively have distributed over a hundred original scripted series (102), signifying a profound shift in the production and distribution of audio-visual content.

### Analyzing diversity on SVOD services

My inquiry focuses on diversity in representation, in terms of on-screen talent and the fictional characters they portray. According to McQuail and van Cuilenburg, the concept of social diversity implies various ‘bases of differentiation’, such as people’s beliefs (political, religious), locality and region, ethnicity, language, social class, gender, life cycle (age), as well as ‘interests, hobbies, skills, and dispositions’ (1983: 148). To this, other axes of analysis should be added, such as bodily and ability diversity. However, as McQuail and van Cuilenburg warn us, adopting a ‘content-focused approach’ to diversity ‘makes heavy demands for data, both quantitatively and qualitatively, which are not easily met’ (1983: 150). This is especially the case when data compilation must be done manually, after carefully watching all productions, as was the case for the present project. For feasibility purposes, my analysis of diversity was therefore restricted to ‘demographic diversity, based on racial, ethnic and gender difference of the people involved in the programme’ (Albornoz and Garcia Leiva, 2019: xxix), while recognizing that social diversity is not limited to these categories.

More generally, the operational definition of diversity employed here acknowledges that it refers to a ‘multiplicity of forms’ (UNESCO, 2005), and an equitable plurality of cultural expressions and identities (Fleras, 2011). This entails a dual-pronged data evaluation. Firstly, with the help of my research team,^5^ I conducted a qualitative and quantitative analysis of non-normative characters, such as those who are non-heterosexual or non-white. Secondly, a thorough exploration of the extent and variety of character profiles and themes in each series' overall narrative has been undertaken. This approach is necessary to prevent a limited view of diversity that would merely tally non-normative characters without fully examining their narrative function in the overall narrative. Limiting diversity to what is socially construed as the ‘Other’ can unwittingly perpetuate the very norms it aims to scrutinize.^6^ Conversely, embracing a definition of diversity as the *plurality of individuals as well as of cultural and identity expressions* allows us to better engage with the political potential of counter-hegemonic representations.^7^

Finally, my assessment of on-screen diversity adopts an intersectional lens, recognizing the complex interplay among various facets of identity and dimensions of oppression/privilege (Brun, 2020; Collins, 2019; Edwards and Esposito, 2019; Harvey, 2020; May, 2015). In practice, this means eschewing a ‘single-axis approach’ to focus instead on *relationality* (Collins, 2019) – the concept that ‘race, gender, class, and other systems of power mutually construct one another’ (16). While it may be necessary to foreground certain aspects of identity when presenting results, an intersectional framework urges us to scrutinize findings by considering the elements of representation that are obscured or overlooked.

An intersectional perspective is particularly useful to remain vigilant in a context where media industries actively promote efforts to enhance on-screen diversity. Such marketing strategies can be misleading, for example, when diversity is promoted through repetitive showcasing of the same character profiles. Employing an intersectional approach thus encourages a more critical stance, prompting us to dissect diversity claims through multiple dimensions of identity concurrently. In that sense, as Vivian M. May reminds us,

“intersectionality provides tools for questioning default explanations about status quo reality and for probing the everyday logics that sustain and rationalize inequality; it is equally useful for identifying gaps between stated goals and actual practices, including unexpected sites of collusion between dominance and resistance” (2015: 6).

The ensuing examination of results therefore incorporates both quantitative metrics^8^ and qualitative observations, all through the lens of intersectional analysis. Yet, before doing so, to better understand the results that will be presented next, it is important to clarify what ‘original content’ means in Canada.

### Defining ‘Original content’ in Canada

In the era of SVOD services, original content is key. Historically, streaming services’ catalogs were predominantly comprised of licensed programs. However, with the proliferation of platforms, SVOD services started prioritizing original content (Lotz, 2022; Lotz and Lobato, 2023). Focusing on ‘original content’ is especially instructive as it sheds light on a streaming service’s curatorial and programming strategies. It offers insights into a platform’s content development ethos – essentially, the brand identity it endeavors to forge and the narrative trends it deems most compelling. As Lotz argues: ‘Distinguishing bespoke content developed specifically for the SVOD is important because services are able to deploy explicit content strategies through these titles’. (2022: 9) Furthermore, because of the specific affordances of internet distribution (Lotz, 2017; Lotz and Lobato, 2023), studying original productions is particularly instrumental in understanding the ‘new kinds of creative possibilities for creators […], [and] modes of storytelling the affordances of SVOD allow’ (Lotz and Lobato, 2023: 4).

Yet, the concept of ‘original content’, often ubiquitously deployed within the media industry, eludes a simplistic definition and warrants further elucidation. As Matthew Ball (2018) contends: ‘“Original” isn’t a technical definition but a marketing one. And thus not only is the definition of “Original” vague, it also differs from network to network’. In academic circles, ‘original content’ is broadly classified into four distinct categories (Afilipoaie et al., 2021; Ball 2018): (1) **developed originals**, that is, bespoke content that is commissioned and produced directly for a streaming service; (2) **acquired originals**, i.e., content that is purchased after production or to resume production; (3) **co-productions** between a streaming service and a national broadcaster; and (4) **licensed originals**, which refer to works not initially made for a streaming service but that have been granted exclusive online distribution rights. While the term ‘original’ can be liberally applied by a streaming company to many different productions, scholarly analysis typically focuses on the first three categories as they more directly reflect a company’s commissioned projects.

While the criteria used to define ‘original’ or ‘bespoke’ content are well-suited for large transnational streaming services (such as Netflix, Amazon Prime Video, Disney+, and AppleTV+) and thus widely adopted by scholars, they do not entirely fit the context of regional or national services. The thread of commonality in the prevailing definitions is the element of *exclusivity*: original content is branded as such when a streaming service secures exclusive distribution rights, whether globally or in specific regions. However, in smaller markets like Canada’s, granting exclusivity to a streaming service might not be realistic. Given the market’s limited size, there is a strategic impetus to diversify broadcast windows, despite an initial promotion as ‘original’ content for a streaming service. Canadian SVODs do not uniformly apply the term ‘original’, and frequently, productions advertised as such are later aired on linear TV channels. For instance, Club Illico offers several series as ‘Originals’, but these typically later find their way to linear TV channels that are owned by the same media conglomerate (Québecor). Hence, the logic of ‘windowing’, though often presented as outdated in the era of streaming (Lotz, 2022: 24), remains a prevailing model in the Canadian media landscape.

Moreover, the Canadian audio-visual sector deeply relies on public funding; even series commissioned by private networks usually benefit from taxpayer subsidies, such as those of the Canada Media Fund. This financial model encourages media companies to maximize broadcasting windows for original content, to enhance the public’s access to works they have collectively invested in. The scenario becomes even more intricate for PSB’s ICI TOU.TV. While the public broadcaster needs to attract subscribers online with ‘original’ content, its mandate to provide universal access to its productions prohibits it from offering scripted series exclusively on its premium service. Consequently, most original scripted series on ICI TOU.TV are branded as ‘ICI Original Series’ (Série originale d’Ici) – a term that encompasses all channels and services of the PSB’s Francophone branch – and are thus ‘premieres’. This means that they debut on the streaming service but are later broadcast on the PSB network. Only a few unscripted shows and short web series – much less expensive than long-form scripted programs – are offered exclusively on ICI TOU.TV.

Hence the notion of ‘original content’ in Canada refers to works exclusively *or* primarily accessible through a local streaming service. This detail is crucial as it illuminates the constraints influencing the commissioning of original content for national SVODs. While producing innovative and progressive content may attract local audiences to these streaming services, there remains a compelling need to ensure that programs and series maintain their appeal to a broader audience, as most will be later broadcast on linear channels. Consequently, this necessitates a delicate balance of production strategies that may appear, in many respects, to be contradictory.

## Results: diversity in French-language Canadian streaming services

### The ‘inclusive pedagogy’ of original French-language series

This study reveals promising developments in the diversification of on-screen characters since 2015,^9^ particularly concerning sexual and gender diversity. Over a third of the 102 scripted series surveyed (37.3%) feature at least one LGBTQ+ character in either a leading or supporting role. Moreover, a remarkable aspect of this inclusivity is the predominance of queer women. The fact that 22.5% of series include a queer woman – a character whose gender and/or sexual identity challenges cisheteronormativity – compared to 17.6% featuring queer men, marks a departure from traditional trends identified in earlier studies. Historically, as highlighted in Quebec (Chanady, 2020, 2022) and international research (Beirne, 2012), there has been an overrepresentation of gay men within queer characters on television. Therefore, such a shift among LGBTQ+ characters signals a meaningful evolution in terms of character development and thematic exploration.

Beyond mere numbers, there is also a qualitative shift progressively taking place in how LGBTQ+ characters are portrayed in original scripted series during this time period. While linear TV has historically relegated them to marginal roles, original series on SVODs tend to craft narratives that foreground and engage with non-normative identities and/or sexualities more substantively. The fact that a series is available on a streaming service thus seems to influence not only casting choices but also story arcs, allowing for a richer exploration of diverse stories.

The trailblazing *Féminin/féminin*, a TOU.TV acquired original series, has already garnered attention in academia (Obermayr, 2019; Ravary-Pilon, 2020). Even though it sparked some critiques for its predominantly white cast and portrayal of conventional themes like marriage and motherhood, it nonetheless stands out as the sole Quebec series centered entirely around a queer community, with all main characters identifying as lesbian or bisexual women. This departs markedly from the usual practice of situating a lone queer character within a heteronormative framework and illustrates how streaming services can foster narrative diversity even within a limited market.

The Quebec remake of the Australian series *Sexy Herpes*, titled *Sans rendez-vous*, which premiered on ICI TOU.TV, is also noteworthy for its candid portrayal of varied gender identities and sexualities within the setting of a sexual health clinic. Significantly, the lead character, Sarah (Magalie Lépine-Blondeau), is a lesbian, marking one of the rare instances where a non-heterosexual character occupies the leading role. The series also broke new ground by introducing a non-binary character, Lou (Mikhail Ahooja), as a key part of the ensemble cast. While the initial season primarily focuses on the popular trope of Lou’s coming-out, subsequent seasons delve more deeply into their journey towards romantic and professional fulfillment. *Sans rendez-vous* also features an asexual character in a recurring role, reflecting a commitment to exploring a broader spectrum of identities and sexualities. The series is particularly notable for its intersectional approach, creating characters that are situated at the intersection of various axes of oppression, as opposed to isolating each form of marginalization.

On teen series, the deliberate inclusion of sexual, gender, and racial diversity takes on even greater significance. Teen dramedies in our corpus frequently engage with feminist discourse, critique established sexual and gender norms, and politicize matters of visibility and recognition. They also often include story arcs that grapple with discrimination, navigate the complexities of sexual violence, and explore the notion of consent. In that sense, it could be argued that many series adopt what Ava Laure Parsemain (2019) calls a ‘queer pedagogy’, that is, a ‘constructivist pedagogical approach, in which the text simply raises problems and questions to invite viewers to “look,” “think” and “engage” with a range of issues’ (8) without seeming to moralize audiences.

For instance, *Les Petits rois* (*Entitled*), a series focusing on a group of high school friends, exemplifies this push towards diverse representation. In addition to showcasing racial diversity, it portrays fluid sexualities that defy strict labels. The narrative also upends traditional stereotypes, featuring characters like Adaboy (Alex Godbout) – a cisgender heterosexual man with a feminine gender expression aspiring to be a fashion designer – and his best friend Julep (Pier-Gabriel Lajoie), a gay man with a sporty look and traditionally ‘masculine’ demeanor. Having been acquired in France, Finland, and Belgium, recognized as a finalist for the Content Innovation Award in 2021, and named the Silver Winner for a drama series at the Telly Awards in 2022, *Les Petits rois* positions itself alongside influential U.S. teen series such as *13 Reasons Why*, *Sex Education*, *Euphoria*, and HBO’s reboot of *Gossip Girl*. This suggests that inclusive narratives championed by U.S. premium services are inspiring similar narrative approaches globally, even persuading funding bodies – such as those integral to Canadian series production – to invest in projects that portray more diverse characters.

Club Illico’s teen series *L'Académie* centers on the tribulations of three teenage girls finishing their secondary school at an elite college, while facing various relational and identity issues. Notably, the series features Wendy (Sabrina Bégin Tejeda), a young Dominican lesbian woman, as one of the main characters. A staunch feminist, Wendy addresses various gender issues, such as sexual consent and the importance of promoting women’s sexual agency, in addition to organizing activist interventions to denounce the homophobia she witnesses on campus. The series also directly tackles the issue of slut-shaming, since two characters are victims of gender-based bullying. At the story’s outset, a new teenage girl (Scarlet) arrives at the Académie after being the target of a widespread bullying campaign at her previous school for kissing a friend’s boyfriend. Later in the series, one of the main characters, Agathe (Léa Roy), ends up in a similar predicament when she is being filmed without her consent during a sexual encounter. When the video is sent to all students, Agathe’s reputation is tarnished. Not only does the series openly criticize such discriminatory behavior, but it also provides moments of dialogue that explicitly state that sharing such videos is a criminal act.^10^

In a context where teen series are still criticized for their heteronormativity (Kaveney, 2006), the ICI TOU.TV original teen drama *Nomades* (*Nomads*) emerges as an even more refreshing deviation. The series breaks new ground by centering on a bisexual teenage girl, Sam (Romane Denis), instead of relegating her to a supporting role. *Nomades* presents her romantic endeavors with both men and women with a subtlety that sidesteps voyeuristic tendencies of the *male gaze* (Mulvey, 1975). Moreover, the series paints a picture of healthy familial dynamics by portraying Sam’s mother as both supportive and understanding of her daughter’s sexuality, thus eschewing the cliché of queerness as a trigger for family conflicts. *Nomades* also stands out by not only showcasing racial but also generational diversity, a notable contrast to the often-homogenized cast of teen series. Indeed, it assigns importance to older women characters that Sam encounters on her journeys, and who become pivotal figures in her life as a burgeoning writer.

In addition to including more diverse characters and adopting a more inclusive narrative approach, series may even briefly resort to a more ‘authoritative’ pedagogical approach. Without adopting an overtly moralizing tone, they sometimes include short scenes where adults’ prejudices are deconstructed by younger characters. For example, *Les Petits rois* features a compelling exchange between Adaboy and his father, who hesitates to discuss his bisexuality with his wife. After Adaboy’s father justifies his discomfort by arguing that people from his generation are less open to sexual ‘difference’, his son replies:

Okay, the world may be more open-minded today, but it’s still not easy for anyone. Whether you’re queer, gay, trans, bi, whatever, it doesn’t matter. You still must be clear with people. First, you need to articulate what you need and then find out if it’s okay with the other person, don’t you think? (ep. 6; my translation)

Similarly, the series *Six degrés* (*Six Degrees*) garnered the Youth Media Alliance’s excellence award for its ‘Commitment, openness to the world, and respect’, and was shortlisted at the MIPCOM 2021 in Cannes for the ‘Diversify TV Excellence Award for Representation of Disability – Scripted’. The show centers around a visually impaired teenager, Léon (Noah Parker), weaving narratives that encompass interracial family dynamics, and a spectrum of body types and sexual identities. It also breaks new ground by portraying one of Léon’s family members, Humberto (Estéban Wurtele), coming out as pansexual and non-binary at the end of the first season. Moreover, *Six Degrés* includes short scenes designed to enlighten characters and viewers alike on the importance of embracing inclusivity and respecting diversity. In such a moment, Humberto’s father Francis (Alexandre Goyette) shares his skepticism with Leon about Humberto’s recent coming-out as pansexual. Léon then intervenes with an enlightening ‘lesson’ to help his father understand that he would have never doubted his kid’s sexuality if they were heterosexual.

**Francis:** Just between you and me ... but Humberto's bisexuality, we agree that he is too young to know that, right?

**Léon:** Pansexuality.

**Francis:** Yeah, *pan*.

**Léon:** And why is he too young to know that?

**Francis:** Because he is eleven years old.

**Léon:** You, when you were 11, did you know if you liked girls more than guys?

**Francis:** Of course!

**Léon:** Well, you got your answer.

**Francis:** Ah! Ah of course, yeah! I am old! Thanks! (season 1, episode 10; my translation)

While maintaining a subtlety that avoids overt moralizing, this type of exchange seems to resort to a form of ‘queer pedagogy’ (Parsemain, 2019) by encouraging viewers to understand queer characters’ perspectives. Through the guise of casual conversation, series skillfully educate and prompt reflection on accepting and understanding difference.

### Streaming racial diversity

Original scripted series on streaming services also showcase greater racial diversity than linear TV. This trend is especially pronounced in a nation like Quebec, which historically faced criticism for its minimal representation of ethnocultural diversity. As early as 2001, Serge Proulx and Danièle Bélanger argued that the representation of immigrant communities on Quebec television was an ‘important socio-political issue’ (119; my translation) since a lack of non-white characters in Quebec series led to a critical disengagement by these communities. The issue of racial diversity, or the lack thereof, has thus been a point of contention in the audio-visual industry for decades. More recently, a 2019 study commissioned by public broadcaster Radio-Canada (Landry, 2019) confirmed that the number of ‘visible minorities’ in prominent Quebec series was increasing, while pointing out that representation is still not proportional to demographics, not to mention that non-white characters rarely occupy leading roles.

Reflecting on the context of Quebec’s media industry, my research indicates a substantial improvement in the representation of racial diversity. An encouraging 66.7% of the 102 scripted series surveyed include at least one non-white character in a recurring role, whether as a lead or supporting character. Notably, 26.5% of original series cast racialized individuals in lead roles, a marked contrast to their previous underrepresentation on linear TV. This figure is particularly significant considering Landry’s (2019) study, which found that only 8.1% of broadcast TV series featured lead actors ‘from a diversity background’ (‘issu de la diversité’). Thus, the present findings suggest a trend toward more inclusive casting practices.

For example, the second season of the short series *Moi, j’habite nulle part* (*I Live Nowhere*) tells the story of Kenza (Leïla Louchem), a woman of Moroccan origin who seeks refuge in a battered women’s shelter, after years of domestic violence at the hands of her white husband. Another groundbreaking series, *Après le déluge* (*Still I Rise*), made a significant impact in 2023 by featuring a predominantly non-white cast for the first time in Quebec television history. This Crave Original has been renewed for a second season. Early 2024 also saw ICI TOU.TV distributing two series with a strong emphasis on cultural diversity: *Ça prend pas la tchas à Papineau* features various characters from the Maghreb, Latin America, and Haiti who live in Montreal, and particularly focuses on Jojo (Lex Garcia), a young widowed father. Competing at Série Mania 2024 in the ‘short form’ category, this series stands out for its contemplative pacing, allowing space for silence and reflection.

On a different note, the comedy *Lakay Nou* provides a humorous glimpse into the life of a Haitian family in Montreal, while challenging many stereotypes. In a particularly sharp scene where the entire family is interviewed by a TV reporter who wants to do a feature on their restaurant, one of the main characters, Myrlande (Catherine Souffront) openly criticizes Canadian diversity policies that ‘put people in boxes’. Incidentally, the scene indirectly criticizes broadcasters’ hypocrisy when they instrumentalize issues of diversity just to obtain public subsidies.

**Reporter:** Could I ask you to fill out these little forms? It’s just a formality.

**Myrlande** (reading the form): ‘A Declaration of Authenticity for Racialized People’? Okay... it’s to prove that we’re authentically Black?

**Reporter:** No, I know, it’s a bit awkward, but it’s a measure to ensure the representation of diversity on screen. The minorities.

[…]

**Myrlande:** I know what diversity means... Do you want us to call our daughter? She’s Black and lesbian.

**Reporter** (looking uncomfortable): Uh no.

**Myrlande:** My parents are Black and wealthy, that’s a ‘minority’. Or me, besides being Black, I’m an honest lawyer. Jackpot diversity! How many points does that get you?

**Reporter:** You know, it’s just that the federal government likes to put everything in boxes, that’s all.

**Myrlande:** Or on reservations. Like the First Nations. (*Lakay Nou*, season 1, episode 8; my translation)

On a different register, Club Illico’s *Le temps des framboises* addresses the issue of migrant workers in the agricultural sector. Far from confining workers to tertiary roles (as is the case with tokenism), the narrative gives them significant visibility. One of these workers, Francisco (Edison Ruiz), even gradually becomes a main character. Thanks to his special friendship with the former owner of the farm, Francisco leads his widow Elizabeth (Sandrine Bisson), the new owner, to become more open to diversity, more sensitive to others, and even to adopt eco-responsible practices.

Club Illico’s *Les Perles (Pearls)*, set against the picturesque Haute-Côte-Nord, is also noteworthy for its inclusion of a recurring Indigenous character, Cynthia, portrayed by Sharon Fontaine-Ishpatao. This character breaks from several stereotypes usually associated with Indigenous people: a cheerful and resourceful young woman, Cynthia shows great emotional maturity and stability, and is a source of comfort for her best friend Stéphanie (Bianca Gervais). Moreover, the series does not shy away from critiquing the main character, Stephanie, a white single mother who struggles to acknowledge her own privilege, which leads her to neglect Cynthia’s emotional needs.

These findings thus align with those of other researchers who have highlighted the potential for racial diversification on streaming services, but also the potential for narrative innovation through the treatment of topics that would be too polarizing for linear television (Martins, 2019).

## Some intersectional observations

**Figure 1.**
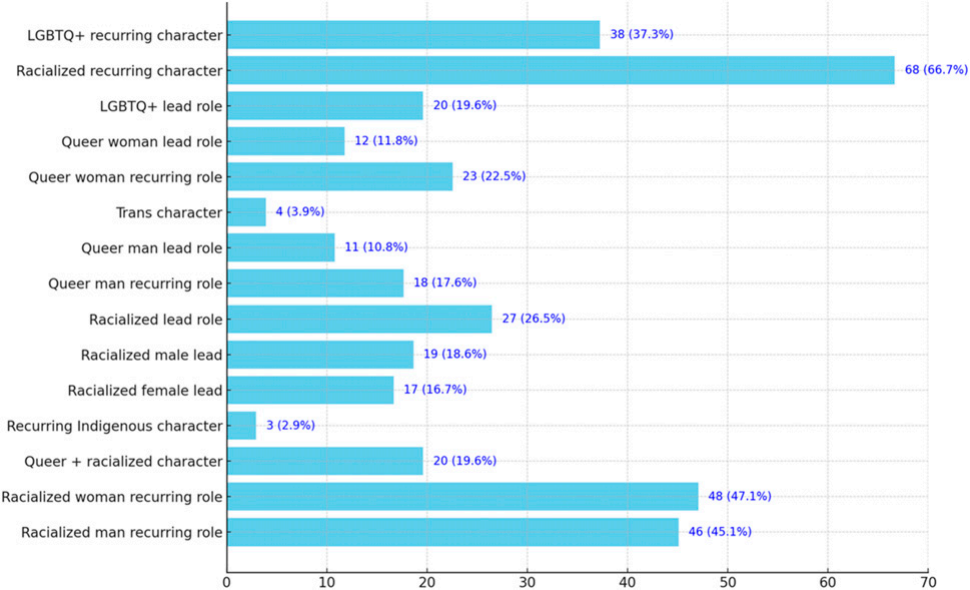
Number of original French-language scripted SVOD series including at least one non-normative character of different categories (2010-2024).

Canadian streaming services distinguish themselves by critically engaging with sexual, gender, and racial norms, setting their original series apart from those produced solely for broadcast networks. The data paints an optimistic picture, indicating a meaningful inclusion of characters who embody various forms of marginalization. Notably, in 19.6% of series, audiences can find at least one character who is both queer and non-white – a figure that could signal a departure from the traditional overrepresentation of white queer individuals, while challenging the dominance of whiteness often characterizing queer spaces (Logie and Rwigema, 2014). Moreover, this intersectional representation is notably more pronounced in teen series, once again demonstrating the central role diversity plays in narratives aimed at younger audiences (Figure 1).

The enhanced inclusion of diverse identities within French-language original scripted series is indicative of a trend noted internationally: streaming services are diversifying content beyond traditional broadcast standards. Scholars have posited that the unique affordances of internet distribution (Lotz, 2017), such as nonlinearity, the greater possibility for narrowcasting and ‘user specificity’ (Lotz, 2017) as well as the potential independence from advertisers, can encourage the production of more diverse and innovative series (Bucciferro, 2019; Christian 2018; Jenner 2018; Khoo, 2023; Martins, 2019; Parsemain 2019). As Amanda D. Lotz summarizes, ‘the aim of creating content that attracts subscribers leads to programming very different than the aim of creating content that will gather a mass of advertiser-desired eyeballs’ (2017: 13). Consequently, according to Aymar Jean Christian, the Internet can bring ‘innovation to television by opening mass distribution to those excluded from legacy development processes, fostering new ways of creating and marketing series’ (2018: 4).

The rise of streaming platforms has also prompted a paradigm shift in the way broadcasters and distributors approach content commissioning and promotion. The much vaguer and normative notion of ‘Quality’ has recently been eclipsed by a focus on diversity. Netflix’s ‘Inclusion & Diversity’ initiative (Khoo, 2023), and its broader commitment to representing diversity to justify its transnational expansion (Asmar et al., 2023; Jenner, 2018: 175) epitomize this industry-wide pivot. Mareike Jenner succinctly observed that Netflix’s advocacy for on-screen diversity ‘includes not only a stronger emphasis on non-white identities, a questioning of heteronormativity, and abroad a variety of series with (often white) female leads, but also multilingualism’. (2018: 174) Moreover, a Nielsen report from 2023 corroborates that streaming platforms outpace linear television in LGBTQ+ representation, underscoring the expansive potential of streaming services. The influence of these production strategies from global/U.S. platforms, which currently dominate the SVOD subscription market in Canada, should thus not be underestimated. Opportunities provided by web distribution and the push for content diversification in a now transnational television industry are contributing factors to the heightened diversity in Canada’s French-language original series.

While recognizing that progress has been made, it is essential to approach these observations with caution. While streaming services hold the potential to transform creative and representational practices, such outcomes are never guaranteed. Moreover, the fact that some streaming services offer more inclusive series does not necessarily mean that they offer equitable representation or that they are free from issues. Therefore, it is crucial to temper optimism with a dose of reality, especially since my analysis underscores persistent disparities in representation. A closer look at lead roles specifically, as opposed to recurring ones, indicates that certain social groups continue to be sidelined as central figures. Close examination of the data reveals that characters who defy hegemonic norms – be they queer, BIPOC, individuals with disabilities, etc. – predominantly occupy peripheral roles. This is particularly true for LGBTQ+ characters, who are still the least represented category in lead roles (11.8% for queer women, 10.8% for queer men), as well as for BIPOC women, who are present in 47.1% of scripted series but only in 16.7% as leading characters. Series also represent very little diversity in terms of capacities and disabilities.

It is also worth pointing out that defining ‘main characters’ in TV series is an intricate task due to the abundance of recurring roles. For this study, a character was deemed central (first role) if they were featured in every episode and involved in numerous storylines, while being highlighted in promotional materials such as posters, articles, and interviews. However, this definition does not always capture narrative significance, as series often consist of an expansive cast where disparities, particularly in speaking time and screen presence, persist. During data compilation, my research assistants and I sometimes sensed that the categorization of BIPOC women as ‘lead’ characters was somehow misleading, as it did not fully reflect their narrative prominence. In other words, their presence within the series sometimes seemed less significant than their male and/or white counterparts. To validate these perceptions, an automated analysis would be necessary to precisely measure each character’s dialogue and screen time.^11^ Currently, due to limited funding for media research in Canada, implementing such IA-driven methods is challenging.

The representation of Indigenous characters in original series is even more limited, with a mere 2.9% featuring at least one. Such a scant inclusion of Indigenous characters underscores an ongoing challenge to authentically reflect Canada’s demographic diversity. However, this issue is not unique to Canada. Gracenote Inclusion Analytics data from Nielsen (2022) also indicates that Native Americans are among the least represented groups in U.S. television series, appearing in only 0.44% of SVOD scripted offerings. Such underrepresentation underscores the need for transnational efforts aimed at devising better reconciliatory and creative measures.

The analysis of LGBTQ+ inclusion in Canadian French-language original series yields a notable trend: gay and lesbian characters still predominate within the broader spectrum of queer representation. Despite notable progress in showcasing sexual and gender diversity, certain identities remain underrepresented. For instance, even though their visibility has grown, trans characters are featured in only a small fraction of series (3.9%), and only one (*Après le déluge*) features a trans character in a lead role. Many series also seem to adopt an ‘assimilationist’ stance towards sexual and gender diversity (Parsemain, 2019: 11), highlighting similarities over differences and promoting the notion that queer people are ‘just like everyone else’. While this approach may of course foster acceptance, it also tends to ‘subdu[e] otherness instead of celebrating it’ (Parsemain, 2019: 11), thus defusing the subversive potential of queer representation. This trend of *gaystreaming* or *homonormalising* (Parsemain, 2019; Rouleau, 2022: 10), previously prevalent on linear TV, persists in the digital realm, indicating that the move to online platforms has not fully upended conventional narrative strategies.

Once again, multiple factors might explain this marginalization of some queer identities and sexualities. On the one hand, the prevalent strategy of windowing in Canada can lead to *multicasting* (Himberg, 2014), which happens when media offerings try to attract specific audiences with non-normative representations, while simultaneously crafting narratives that appeal to a more general audience. By foregrounding gay and lesbian characters as emblems of ‘sexual and gender diversity’, series can thus engage niche audiences without alienating a general audience that may not fully grasp the entire spectrum represented by the LGBTQIA+ acronym. Additionally, the tendency to assign LGBTQ+ characters to supporting roles might serve to create an inclusive story, while still endorsing a normative social order where queer identities and sexualities are seen as secondary or ‘less normal’ than others.

On the other hand, while streaming services like Netflix promote themselves as way more inclusive than traditional broadcast networks (WeAreNetflix, 2023), they are not immune to critique. A persistent issue is that LGBTQ+ characters often occupy supporting rather than central roles in their original series (Boisvert, 2019b). Another is that this promotion of diversity still relies on social norms, thus not really challenging hegemonic discourses that initially led to the marginalization of certain groups (Arnold, 2016; Jenner, 2018): ‘“Netflix” diversity still sets white, male, able-bodied, middle-aged, middle-class and heterosexual as “the norm” while everybody else is “diverse.”’ (Jenner, 2018: 176) Aymar Jean Christian also notes that major streaming giants grapple with representation, since their desire to reach the largest number of subscribers ultimately leads them to tamper the inclusive and innovative scope of their series: ‘Even networked TV distributors like Netflix, presumably able to target a diverse base of users through algorithms, struggle with representation. In their pursuit of “big data,” networked distributors are replicating the inequalities of the legacy media era, privileging bigger audiences over smaller, intersectional ones’ (2020: 462).

Moreover, according to Campion (2019), streaming giants’ desire to secure online hegemony gradually leads them to resort to production methods traditionally associated with broadcast networks. Seen in this light, the *qualitative marginalization* of LGBTQ+ characters that my research project highlights extends way beyond Canadian borders, signaling a pervasive, transnational issue of ‘precarious diversity’ within corporate media (Gray, 2016 quoted in Smith et al., 2019: 124). Simply put, as companies grapple with representation, they often struggle to dismantle the entrenched ‘matrixes of domination’ (Christian 2020: 460; Collins, 2000) that have ensured their profitability.

## Diversity as ‘public service’: The contribution of PSBs to the diversification of series on streaming services

For this article, data from three distinct Canadian streaming services – ICI TOU.TV, Club Illico, and Crave – have been synthesized, to offer a comprehensive overview of original French-language content production on Canadian streaming services, and thus assess the level of *external diversity* (McQuail and Van Cuilenburg, 1983), that is, the plurality of representations at the meso level of the Francophone streaming industry in Quebec and Canada. However, this broad approach has limitations as it hides disparities between SVODs in terms of their diversity. Notably, the public streaming service ICI TOU.TV seems to outperform its private counterparts by featuring a more diverse range of protagonists, encompassing a higher number of queer and non-white characters. More specifically, although comparing the percentages of representation from one service to the other would be problematic (as the number of original scripted series varies too much^12^), it appears that ICI TOU.TV offers a significantly larger number of series including more than one non-normative (non-white, non-cis, and/or non-heterosexual) character (15 scripted series, 26.3%). This means that non-normative characters occupy a larger proportion of roles within a same series, which contrasts with the popular practice of isolating a so-called ‘diversity’ character within an otherwise relatively homogeneous cast.

This observation deserves emphasis, considering that the expansion of online television has challenged the public service broadcasting (PSB) model. In particular, the dominance of global streaming giants – all private entities – has led to the popularization of financing mechanisms (such as the subscription model) that do not align well with public service media (PSM). Moreover, as McElroy and Noonan argued,

New empowered actors are accumulating valuable resources (especially data, advertising revenue and attention) and exercising gatekeeping power to the detriment of less powerful players that lack the necessary resources to compete successfully. The pervasive logic of neoliberalism has not only reduced the regulation of global media organisations, but helped drive down public funding. (2017: 171)

Consequently, the present study suggests that public broadcasters may be more inclined to deliver diverse content – or at least to contribute significantly to a diversification (both quantitative and qualitative) of scripted series – since they are driven by considerations beyond mere popularity or profitability. Despite the pressures of the digital era, which include the necessity to boost subscription numbers, public broadcasters face the imperative to devise monetization strategies that are compatible with their public service mandate, such as representing the full diversity of the population. Moreover, public broadcasters typically dedicate a larger part of their budget to commissioning original content (McElroy and Noona, 2017), which may lead to a broader variety of stories being told. The commitment of public broadcasters to programming for children and teenagers – a demographic they need to serve and represent – can also result in more inclusive and novel representations (Andersen and Sundet, 2019). As the international success of the Norwegian *SKAM* format illustrates, a public broadcaster can influence narrative and representational approaches in the digital era because of its mandate to innovate and connect more effectively with local audiences. More broadly, PSMs have a duty to foster local content, to encourage creative expression, and to serve as a safeguard against the concentration of media ownership (Tremblay et al., 2019) – commitments that can lead to the production of more varied content.

Since streaming services discussed in this article provide content in French, a language that holds minority status in Canada, it also becomes obvious that public broadcasters, by promoting local content, contribute to the linguistic diversity of audio-visual offerings. Indeed, while commercial streaming services Club Illico and Crave feature original French-language films and series, their promotional strategies mostly prioritize foreign English-language content. Crave thus mainly owes its popularity to its exclusive distribution rights of HBO, HBO Max and Showtime series in Canada, while Club Illico stands out by offering many Hollywood series and films dubbed in French. Therefore, private SVOD services adopt the ‘Hollywood-hits-plus-local-originals strategy’ (Mike Sneesby quoted in Lobato et al., 2023: 43), which means that original productions play a supplementary role to foreign content. On the contrary, local content is the cornerstone of ICI TOU.TV’s catalog, thus highlighting a different approach to curation and promotion.

As McElroy & Noonan argue: ‘Reducing PSM to a tool merely to plug holes caused by market failure in commercial media not only diminishes the diverse cultural, social, and economic values of their offerings, but also excludes the distinct role that minority-language PSB organizations play in ensuring linguistic vibrancy and diversity’ (2017: 171). Given that public service broadcasters face threats to their very existence, the results of this research prompt a reconsideration of the value of public streaming services in the digital era. Due to their unique mandates and objectives, PSBs might play a significant role in fostering diversity and formulating innovative storytelling methods.

## Conclusion

The objective of this article was to summarize findings regarding diversity in French-language scripted original content produced in Canada, while contributing to a better understanding of the *storytelling affordances* of national/regional SVOD services, that is, these services’ potential or capacity to favor distinct narrative and aesthetic practices, given their specific modes of distribution, financing, and audience targeting (even though their accessibility is still nationally limited). In so doing, this research underlined a noteworthy expansion in the representation of sexual, gender, and racial diversity, as well as a narrative tendency to tackle oppression through an intersectional lens. These findings, consistent with other scholarly work, showcase the capacity of SVOD services and public service media to foster more inclusive storytelling. Yet, despite these advancements, data reveals that considerable work remains to be done in order to truly diversify serial productions and address the *qualitative marginalization* of various social groups. Consequently, such outcomes suggest the need for a more critical evaluation of the purported ‘progress’ or ‘revolution’ in representations offered by online-distributed television (Lotz, 2018).

Moreover, it is crucial to acknowledge the limitations of this research. While establishing statistics on inclusive representation helps understanding narrative trends, it is important to note that calculations have limited value, given the overall low number of French-language scripted series on Canadian streaming services. With only 102 original scripted series surveyed, percentages appear disproportionately high as each additional representation significantly impacts the overall numbers. Since calculations were performed manually following extensive notetaking, the analysis was based on series, as a unit of analysis, rather than on individual characters. This means that statistics emphasize the percentage of series that include various categories of characters, rather than how each category is represented within the total number of recurring characters in each series. This approach may lead to an overestimation of non-normative characters, such as in scenarios where a racialized character shares the lead with several white characters, or where a LGBTQ+ character is featured alongside many cishet friends or colleagues. Yet, accurately calculating the representation of each character category within the total number of recurring characters would require complex, time-consuming computations that are nearly impossible to manage without automated analysis methods. Therefore statistics must be framed by qualitative data. As Jane Ritchie argues, sometimes ‘the phenomenon is too complex or delicate to be captured fully in statistical enquiry and qualitative research is needed alongside to provide the detail or understanding that is required’ (2003: 41). In that sense, calculations presented in this article remain valid, but must be considered in conjunction with the textual analysis to achieve a well-contextualized understanding that incorporates descriptive, explanatory, and evaluative elements.

It is also important to emphasize that this article focuses only on *on-screen diversity*. Further research is therefore needed to provide a more comprehensive picture of diversity inclusion within the Canadian and Quebec streaming industries. Initial findings reveal promising trends, such as near parity in screenwriting roles, but also persistent issues like the predominance of white men in directorial roles. Given the Canadian norm of a series being directed by a single individual – thereby heightening the role’s cultural legitimacy – the fact that a vast majority of series have been directed by white men needs to be questioned. It also echoes similar disparities that have been observed in the United States regarding the low percentage of audio-visual productions directed by women, as well as the alarmingly low rate of Hollywood top-grossing films directed by BIPOC women (Lang, 2023).

According to Aymar Jean Christian, it is on this point that ‘indie’ TV channels prove particularly beneficial, since ‘the value of intersectionality is most visible in *production* (i.e., supporting new writers and shows) and *reception* (i.e., cultivating new fans for them)’ 2020: 460). However, this raises a critical question: in an ecosystem dominated by a handful of transnational streaming services, how feasible is it to launch an indie platform in a non-English language? The economic instability facing many Francophone media players, even leading some to close down – such as the ‘French Netflix’ SALTO (2019-2023) – highlights the urgent need for research on the diversity of representation, yes, but also on the diversity of streaming services themselves. Research is crucial to better understand the systemic challenges affecting platform diversity, particularly for services grappling with compounded issues of cultural and linguistic marginalization.

## Notes

1 A clarification is necessary regarding the ‘origin’ of scripted series discussed in this article. The French-language original series will be described here as ‘Canadian’, since streaming services distributing them are available across the country. However, Quebec is the only Canadian province with a majority Francophone population. Consequently, most of the series discussed here have been produced in Quebec and are locally defined as ‘Quebec series’. Audiences watching them are also mostly located in Quebec. However, these series are not exclusively intended for the Quebec audience, as they may also interest Francophone communities living outside the province.

2 Such an oversight of Francophone production extends globally. Even studies conducted in France tend to privilege U.S. platforms and series (Baldacchino, 2019; Cousin, 2018; Berton, 2016; Perticoz, 2019), given their cultural legitimacy.

3 The very first original Francophone web series, *En audition avec Simon*, debuted on ICI TOU.TV on March 1, 2010. Consequently, the scope of this research encompasses all original Francophone content distributed across the three specified SVODs, starting from the inception of *En audition avec Simon* in 2010 through to February 1, 2024, the date when this article was completed. It is important to note, however, that following the release of *En audition avec Simon*, no other original scripted productions were distributed until 2015.

4 The scripted series were specifically chosen to encompass a wide range of genres, including teen series, dramas, comedies, anthology series, and science fiction, among others. This selection strategy was employed to capture a broad spectrum of themes and aesthetic approaches utilized in the original series, thereby facilitating a more comprehensive analysis of prevailing serial trends. As for the other scripted series that were not subject to detailed textual analysis, they were still partially viewed, typically one or two episodes, to identify the sociodemographic profiles of recurring characters, thus enabling their inclusion in statistical calculations.

5 I would like to sincerely thank my three assistants whose exceptional involvement and rigorous work have been invaluable for this research project: (Dominique Gagnon, Juliette Lavallée and Théo Boisvert).

6 According to this view, white characters would be perceived as ‘normal’ or ‘by-default’ characters, while non-white characters would be defined as ‘diverse’.

7 For instance, a series including a higher percentage of LGBTQ+ characters than their demographic proportion in real life could be interpreted, not as ‘inaccurate’, but as subverting cisheterosexual norms.

8 The statistical enquiry presented in the rest of this article is based on the total number of scripted series (102) identified on ICI TOU.TV, Club Illico and Crave. Detailed textual analysis of approximately half of these series enriched our findings, providing deeper insights into character development and narrative construction. These calculations, accurate as of February 1, 2024, were meticulously performed and cross-verified by three members of the research team. It is pertinent to note that these statistics are subject to change, as new original productions are continually added to these platforms.

9 Although original scripted series were produced prior to that year, 2015 seems to mark a turning point for LGBTQ+ representation with the arrival of *Féminin/féminin* on ICI TOU.TV, a dramedy centered on the lesbian community in Montreal.

10 For a comprehensive analysis of this series, see (Boisvert et al., 2024).

11 For instance, the Gena Davis Inclusion Quotient (GD-IQ) of the Gena Davis Institute on Gender in Media provides detailed statistics regarding ‘intersectional on-screen representation’ in Hollywood movies.

12 Since the number of scripted series varies greatly from one service to another (57 for ICI TOU.TV, 27 for Club Illico, and 18 for Crave as of February 1, 2024), it is impossible to make meaningful comparisons based on percentages of representation. Given their lower number of series, percentages are, in many cases, higher for Club Illico and Crave. It would be presumptuous (or at least premature) to conclude that there is a greater inclusion of certain categories of characters on these commercial streaming services.
